# Supplementary material for: Modeling Stratum Corneum Swelling for the Optimization of Electrode-Based Skin Hydration Sensors
Source: Sensors (Basel). 2021 Jun 9;21(12):3986. doi: 10.3390/s21123986 (PMC8229638; doi:10.3390/s21123986)
Supplement: Supplementary file 1 [file sensors-21-03986-s001.zip › sensors-1204060-supplementary.pdf]

Supplementary Information: Simulating the Electrical Properties of the Human Skin for the Development of Hydration Sensors

Claudio Malnati, Daniel Fehr, Mathias Bonmarin

February 28, 2021

S1 Dielectric Spectrum

The dispersive dielectric spectra of the three skin layers, stratum corneum (SC), epidermis/dermis (E/D), and hypodermis (HYP), are illustrated in figures S1 and S2. The dielectric parameters are taken from [3] for the SC layer and from [1] for E/D layer as blood and HYP layer as infiltrated fat. The different dielectric properties are clearly visible. The dielectric parameters have different magnitudes and the dispersions occur at different frequencies.

S2 Linear Fit of Stratum Corneum Dielectric Properties

S. Naito et al. provides measurements of the SC at three ambient humidity levels; 40%, 60%, and 80% relative humidity [2]. The SC water concentration for these three measurements was simulated using the SC hydration model. First, the water content was initialized as fully hydrated and the skin surface exposed to 30% relative humidity. Once the steady state was reached, the skin surface was exposed to the three ambient humidity levels for 30 min to reproduce S. Naito's measurement procedure. In all three cases, the simulation reached equilibrium after 30 min. This justifies to use the data provided in [2].

With only three sample points per dielectric parameter, it is difficult to investigate the relationship with the water concentration. S. Naito observed that the dielectric properties of free water in SC behave linear in two ranges of water concentration. Range 1 above a water concentration of $0.45\,\mathrm{g/cm^3}$ and range 2 below. The simulations of the SC hydration model result in water concentration of $0.592\,\mathrm{g/cm^3}$ for 40%, $0.598\,\mathrm{g/cm^3}$ for 60%, $0.619\,\mathrm{g/cm^3}$ for 80% ambient relative humidity. All results are above a water concentration of $0.45\,\mathrm{g/cm^3}$. Therefore, a linear fit is appropriate, as also claimed in [2].

The fit was performed with linear regression (1).

$$\vec{y} = \boldsymbol{X}\vec{\beta}, \tag{1}$$

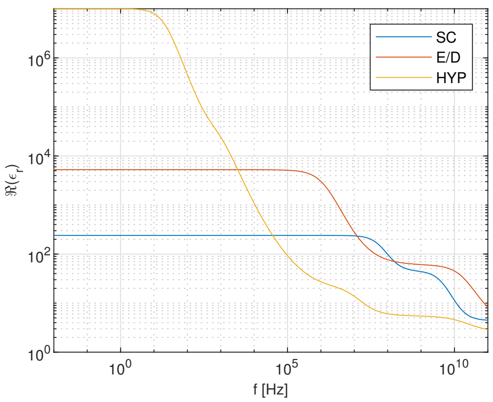

Figure S1: Real part of dispersive dielectric spectrum for the three skin layers. The X axis displays the frequency in [Hz] and the Y axis the real part of the relative permittivity. Each skin layer shows different dielectric behavior. The dispersive regions are indicated by the drop magnitude at several frequencies.

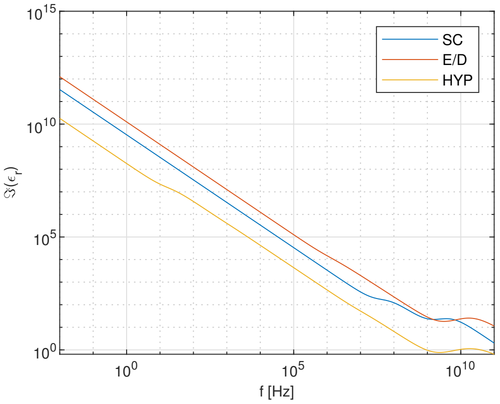

Figure S2: Imaginary part of dispersive dielectric spectrum for the three skin layers. The X axis displays the frequency in [Hz] and the Y axis the imaginary part of the relative permittivity. Each skin layer shows different dielectric behavior. The dispersive regions are indicated by the local maxima in magnitude occurring at several frequencies.

where $\vec{y}$ is the sample vector, $\boldsymbol{X}$ is the design matrix, and $\vec{\beta}$ is the parameter vector. Applying the linear regression to the dielectric parameters, the equation becomes:

$$\vec{y} = \beta_0 + \beta_1 \vec{C}_w, \tag{2}$$

with $\vec{y}$ as the sample vector representing the dielectric parameters, $\vec{C}_w$ is the vector of water concentrations at measurement of the dielectric parameters, and β_0 and β_1 are the parameters to be estimated. Because no samples are provided in the lower hydration range, the linear fit resulted in a scaling of the water concentration C_w with β_0 set to 0. The dielectric parameters can be interpolated with the estimated parameters β_1.

$$\Delta\hat{\varepsilon}_{free} = \beta_{\Delta\varepsilon_{free}} C_w(a_w), \tag{3}$$
$$\hat{\tau}_{free} = \beta_{\tau_{free}} C_w(a_w), \tag{4}$$
$$\Delta\hat{\varepsilon}_{slow} = \beta_{\Delta\varepsilon_{slow}} C_w(a_w), \tag{5}$$
$$\hat{\tau}_{slow} = \beta_{\tau_{slow}} C_w(a_w), \tag{6}$$

where each dielectric parameter has their own scaling parameter β. $\Delta\hat{\varepsilon}_{free}$, $\hat{\tau}_{free}$, $\Delta\hat{\varepsilon}_{slow}$, and $\hat{\tau}_{slow}$ are the interpolated dielectric parameters. Finally, the SC relative permittivity

can be estimated from the Cole-Cole model

$$\hat{\varepsilon}_r = \hat{\varepsilon}_\infty + \frac{\Delta\hat{\varepsilon}_{free}}{1 + (j\omega\hat{\tau}_{free})} + \frac{\Delta\hat{\varepsilon}_{slow}}{1 + (j\omega\hat{\tau}_{slow})}. \tag{7}$$

With equation (8), the dielectric parameters can be formulated in dependency of the water activity a_w.

$$C_w(a_w) = \frac{V_m c k \rho_{mem} a_w}{1 + (V_m c k \frac{\rho_{mem}}{\rho_w} - 2k + ck) a_w + (k^2 - ck^2) a_w^2} \tag{8}$$

The estimated relative permittivity spectrum in dependency of the water activity is illustrated in figure S3.

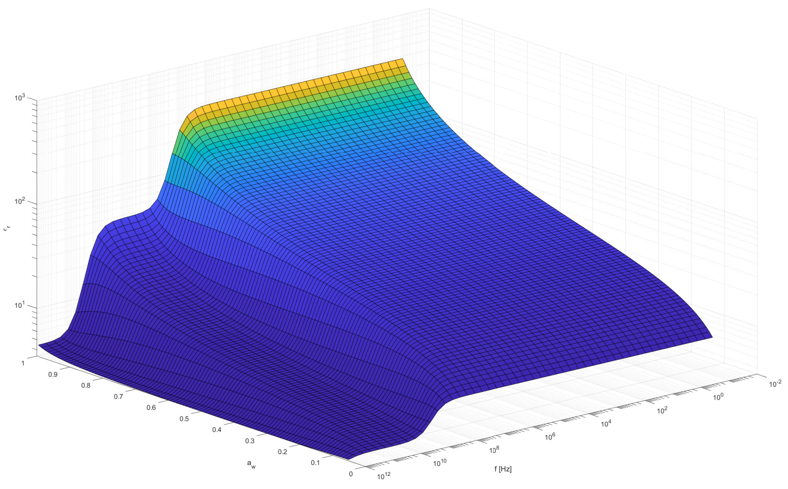

Figure S3: Estimated SC dielectric spectrum. The X axis shows the water activity a_w, the Y axis the frequency in [Hz], and the Z axis the estimated SC relative permittivity $\hat{\varepsilon}_{free}$.

S2.1 Non-Linear Fit

S. Naito et al. mentioned two linear ranges of the relationship between the dielectric parameter of free water and the water concentration in SC with the border $0.45\,\mathrm{g/cm^3}$. Extending the ε_{free} samples from [2] with the two boundary points of 3.3 for completly dry tissue (polymere in S. Naito's assumption) and 72.3 for pure water, the fit can be more

complex estimating four parameters. Then the equation of the water concentration can be fitted to the free water permittivity as follows:

$$\hat{\varepsilon}_{free} = \beta_0 + \frac{\beta_1 V_m ck\rho_{mem} a_w}{1 + \beta_2(V_m ck\frac{\rho_{mem}}{\rho_w} - 2k + ck)a_w + \beta_3(k^2 - ck^2)a_w^2}, \quad (9)$$

where β_0, β_1, β_2, and β_3 are the fit parameters to be estimated. Now the relationship is no longer linear and shows a behavior explaining the two ranges observed by S. Naito et al., as shown in figure S4.

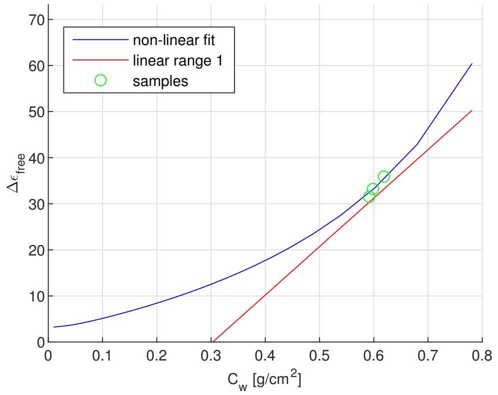

Figure S4: Non-linear fit of ε_{free} according to (9). The X axis shows the water content in [g/cm^3] and the Y axis ε_{free}. The blue line indicates the non-linear fit of ε_{free}, the red line the linear range 1 observed in[2], and the green circles mark the samples also from [2].

S3 Frequency Analysis

This section analyzes the dielectric behavior at specific frequencies for the three fringing-field electrode types: circular impedance electrode, interdigitated impedance electrode, and interdigitated capacitance electrode. In figures S5 - S8 for all electrodes, a trend is visible of increasing capacity for lower water activity $a_w < 0.95$ and a decrease at higher water activity $a_w > 0.95$. Further, the capacity magnitude decreases with increasing frequency.

The main part of the resulting capacity is due to the electric field penetrating into the E/D layer, due to higher relative permittivity, see figure S1. Due to the SC hydration model, the SC thickness increases with increasing water activity. The thicker the SC, the less deep the electric field penetrates into the E/D layer. The dielectric hydration model is responsible that the relative permittivity increases as well with increasing water activity. Now, combining these three effects, the trend of increasing and decreasing capacity depending on the water activity can be explained. For lower water activity $a_w < 0.95$, the

capacity increase due to the SC permittivity is stronger than the decrease due to SC thickness. Thus, increasing water activity brings an increase in capacity. The SC swelling is nonlinear and the thickness increases 65% in the higher water activity range of $a_w > 0.95$. In that range, the reduction of the capacity due to SC thickness is stronger than the increase from permittivity. Therefore, a decrease in capacity is observed compared to lower water activity.

The magnitude difference between frequencies can again be explained with the dielectric spectrum in figure S1 and S2. For frequencies below 1 MHz, the dielectric parameters of the SC and E/D layer are constant in frequency. The HYP layer is highly dispersive at these frequencies. However, its influence is almost negligible because the electric field is very weak at that depth, especialy for the interdigitated electrodes due to their geometry. Therefore, in lower frequencies <1 MHz, there is not much change in magnitude. At higher frequencies >1 MHz however, the dispersive effects occur and the relative permittivity of the SC and E/D layers drop significantly.

Only at frequencies of 100 MHz, the trend of increasing/decreasing capacity is not visible, see figures S6 and S8. Again looking at figure S1, this is the only frequency in this simulation where the permittivity of the E/D layer drops below the permittivity of the SC layer. The capacity increase/decrease compensation of the SC thickness and SC dielectric parameter has a stronger weight and results in a different trend of the resulting capacity.

S4 Sensitivity Analysis

The sensitivity analysis is performed at different SC hydration levels with water activity of $a_w = 0.8$ to 0.996 at 1MHz. The analysis is performed on the three electrode types; circular impedance electrode, interdigitated impedance electrode, and interdigitated capacitance electrode. For each electrode, the absolute and the relative capacitive sensitivity was calculated with the equations (10) and (11) in relation to the simulated capacity at fully hydrated level of $a_w = 0.996$.

$$C_{abs} = C_{a_w} - C_{0.996}, \tag{10}$$

$$r = \frac{C_{a_w} - C_{0.996}}{C_{0.996}} \tag{11}$$

where C_{a_w} is the simulated capacity in [F] at specific water activity a_w, $C_{0.996}$ is the simulated capacity in [F] at water activity $a_w = 0.996$, C_{abs} is the absolute sensitivity in [F], and r is the relative sensitivity.

The influence of the electrode dimensions is investigated by varying the electrode's size and the gap between the electrodes. The areas of the driving and sensing electrode are always kept equal.

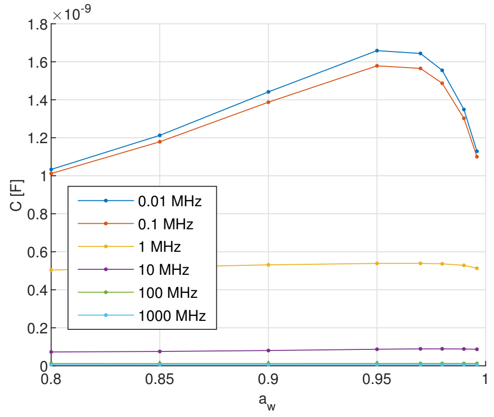

Figure S5: Capacity simulation of the circular impedance electrode at logarithmically spaced frequencies from 0.01 MHz to 1000 MHz. The X axis shows the water activity a_w and the Y axis displays the capacity C in [F]. The dimensions of the skin are for epidermis/dermis 1 mm and for hypodermis 2 mm. The dry SC thickness is set to 9.55 µm.

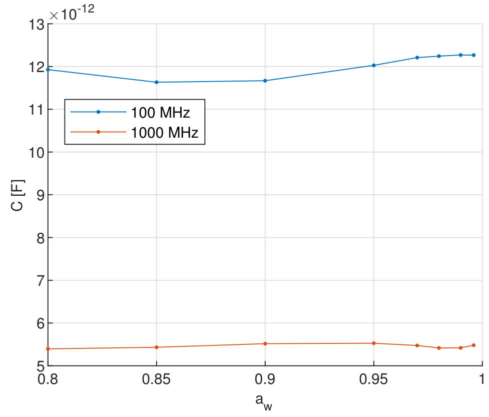

Figure S6: Zoom into the higher frequencies 100 MHz and 1000 MHz of the circular impedance electrode from figure S5.

S4.1 Circular Impedance Electrode Analysis

Figure S11 and S12 show the absolute and relative sensitivity with varying electrode gap and fixed electrode size/area. It is visible that with shrinking electrode gap, the sensitivity increases.

Figure S13 and S14 show the absolute and relative sensitivity with varying electrode size and fixed electrode gap. Also here is visible that reducing the electrode size increases the sensitivity.

S4.2 Interdigitated Impedance Electrode Analysis

Figure S15 and S16 show the absolute and relative sensitivity with varying electrode gap and fixed electrode size/area. The simulation results show an optimum in relative sensitivity at an electrode gap of 0.12 µm. In the absolute sensitivity, the optimum is can also be identified.

Figure S17 and S18 show the absolute and relative sensitivity with varying electrode width and fixed electrode gap. It is visible that with increasing electrode width the sensi-

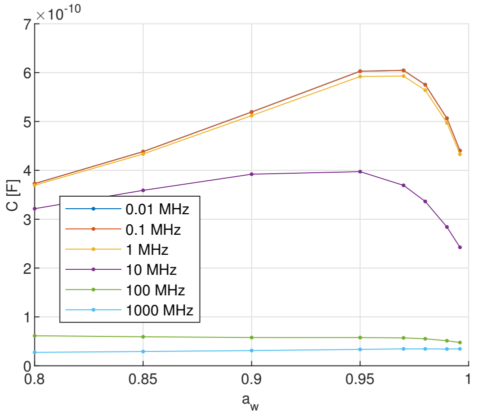

Figure S7: Capacity simulation of the interdigitated impedance electrode at logarithmically spaced frequencies from 0.01 MHz to 1000 MHz. The X axis shows the water activity a_w and the Y axis displays the capacity C in [F]. The dimensions of the skin are for epidermis/dermis 1 mm and for hypodermis 2 mm. The dry SC thickness is set to 9.55 µm.

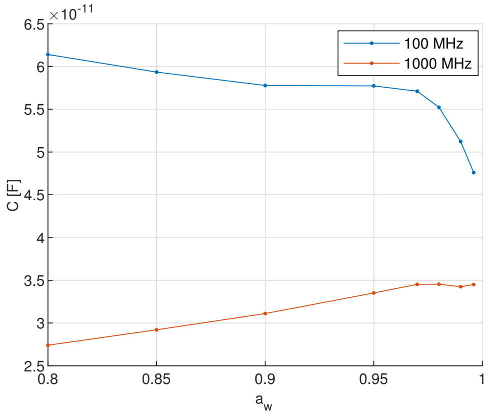

Figure S8: Zoom into the higher frequencies 100 MHz and 1000 MHz of the interdigitated impedance electrode from figure S7.

tivity increases as well. However, the relative sensitivity indicates a converging trend.

S4.3 Interdigitated Capacitance Electrode Analysis

Figure S19 and S20 show the absolute and relative sensitivity with varying electrode gap and fixed electrode size/area. At high hydration level $a_w > 0.95$, the difference in sensitivity is much smaller than in the lower hydration levels. Highest sensitivity shows the largest electrode gap in that range. At lower hydration level $a_w < 0.95$, the wider electrode gaps are also more sensitive. However, the sensitivity fluctuates and no optimum or convergence can confidently be determined.

Figure S21 and S22 show the absolute and relative sensitivity with varying electrode width and fixed electrode gap. The same trend is visible as with varying electrode width d. At high hydration level $a_w > 0.95$, the difference in the sensitivity is bigger for wider electrodes. At lower hydration level $a_w < 0.95$, the trend is an increase in sensitivity the smaller the electrode width.

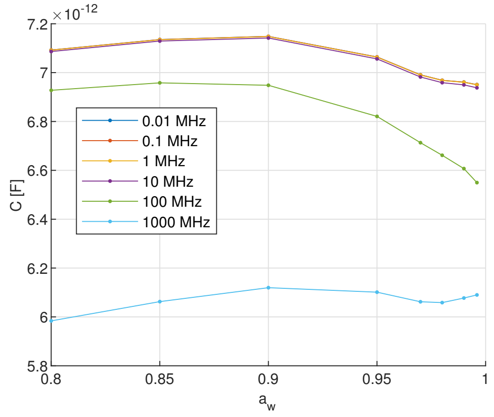

Figure S9: Capacity simulation of the interdigitated capacitance electrode at logarithmically spaced frequencies from 0.01 MHz to 1000 MHz. The X axis shows the water activity a_w and the Y axis displays the capacity C in [F]. The dimensions of the skin are for epidermis/dermis 1 mm and for hypodermis 2 mm. The dry SC thickness is set to 9.55 µm.

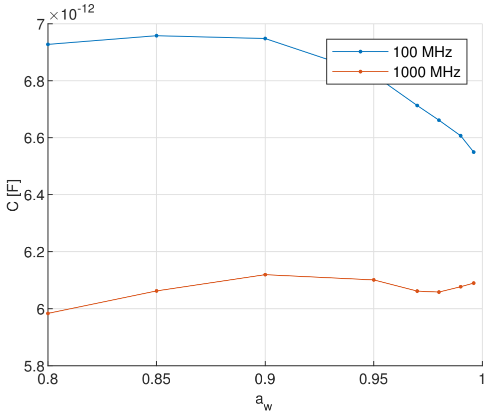

Figure S10: Zoom into the higher frequencies 100 MHz and 1000 MHz of the interdigitated capacitance electrode from figure S9.

References

[1] S Gabriel, R W Lau, and C Gabriel. The dielectric properties of biological tissues: III. parametric models for the dielectric spectrum of tissues. page 25.

[2] Satoru Naito, Masato Hoshi, and Satoru Mashimo. In VivoDielectric analysis of free water content of biomaterials by time domain reflectometry. 251(2):163–172.

[3] Satoru Naito, Masato Hoshi, and Shin Yagihara. Microwave dielectric analysis of human stratum corneum in vivo. 1381(3):293–304.

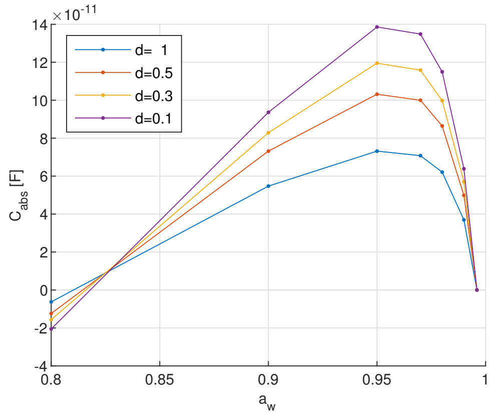

Figure S11: Absolute sensitivity with varying electrode gap of the circular impedance electrode at 1 MHz. The X axis shows the water activity and the Y axis the resulting absolute sensitivity C_{abs}. d is the electrode gap between driving and sensing electrode. The radius of the sensing electrode was kept at 2.5 mm.

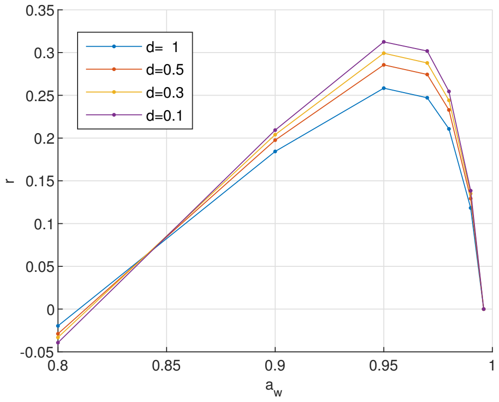

Figure S12: Relative sensitivity with varying electrode gap of the circular impedance electrode at 1 MHz. The X axis shows the water activity and the Y axis the resulting relative sensitivity r. d is the electrode gap between driving and sensing electrode. The radius of the sensing electrode was kept at 2.5 mm.

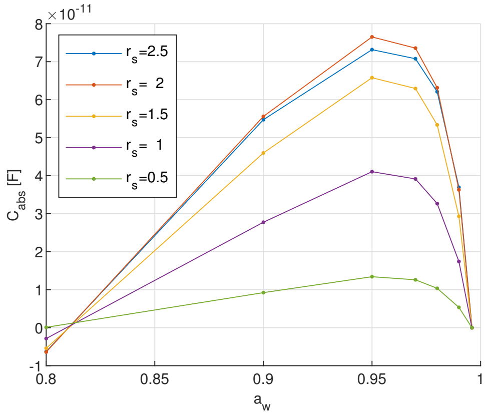

Figure S13: Absolute sensitivity with varying electrode size of the circular impedance electrode at 1 MHz. The X axis shows the water activity and the Y axis the resulting absolute sensitivity C_{abs}. r_s is the radius of the sensing electrode. The electrode gap was kept at 1 mm The driving electrode dimensions are calculated to result in the same area as the sensing electrode area.

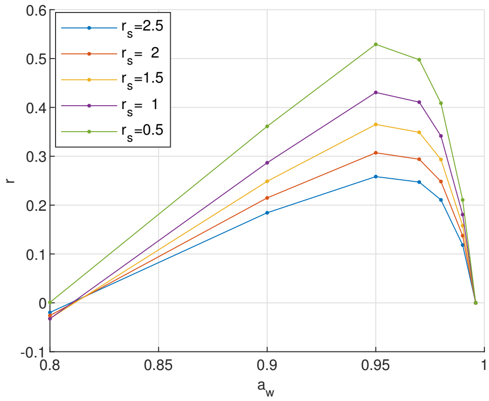

Figure S14: Relative sensitivity with varying electrode size of the circular impedance electrode at 1 MHz. The X axis shows the water activity and the Y axis the resulting relative sensitivity r. r_s is the radius of the sensing electrode. The electrode gap was kept at 1 mm. The driving electrode dimensions are calculated to result in the same area as the sensing electrode area.

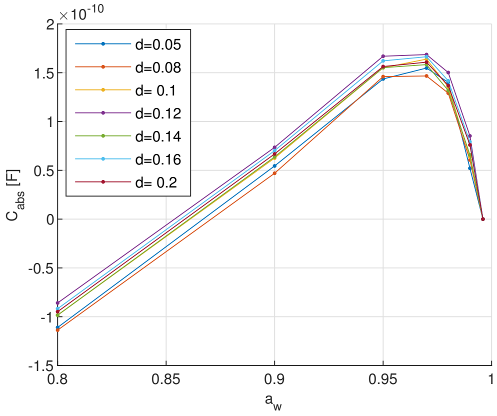

Figure S15: Absolute sensitivity with varying electrode gap of the interdigitated impedance electrode at 1 MHz. The X axis shows the water activity and the Y axis the resulting absolute sensitivity C_{abs}. d is the electrode gap between the electrode fingers. The electrode finger width was kept at 0.1 mm.

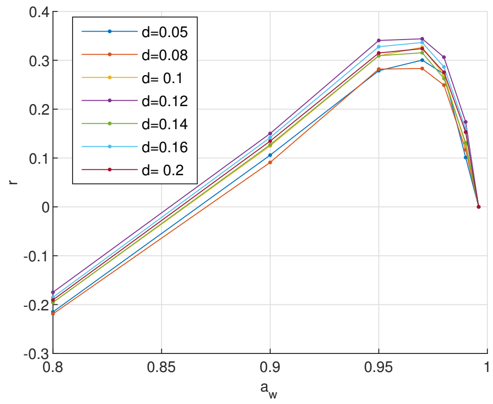

Figure S16: Relative sensitivity with varying electrode gap of the interdigitated impedance electrode at 1 MHz. The X axis shows the water activity and the Y axis the resulting relative sensitivity r. d is the electrode gap between the electrode fingers. The electrode finger width was kept at 0.1 mm.

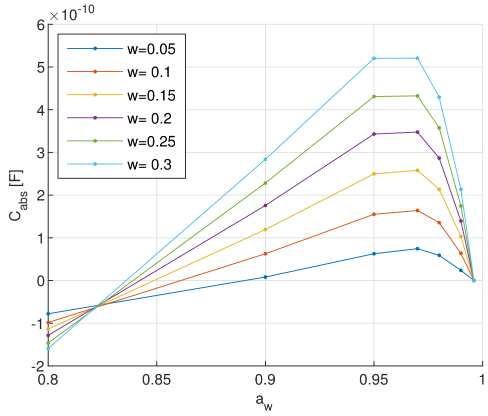

Figure S17: Absolute sensitivity with varying electrode size of the interdigitated impedance electrode at 1 MHz. The X axis shows the water activity and the Y axis the resulting absolute sensitivity C_{abs}. w is the electrode finger width. The electrode gap was kept at 0.1 mm.

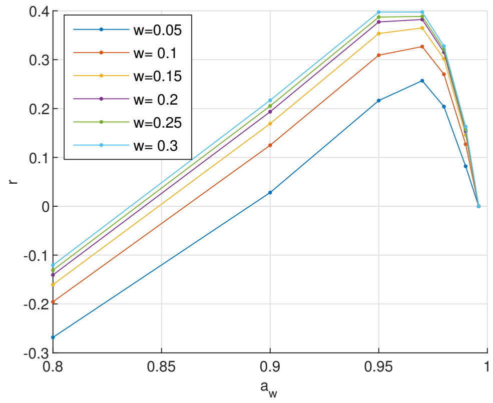

Figure S18: Relative sensitivity with varying electrode size of the interdigitated impedance electrode at 1 MHz. The X axis shows the water activity and the Y axis the resulting relative sensitivity r. w is the electrode finger width. The electrode gap was kept at 0.1 mm.

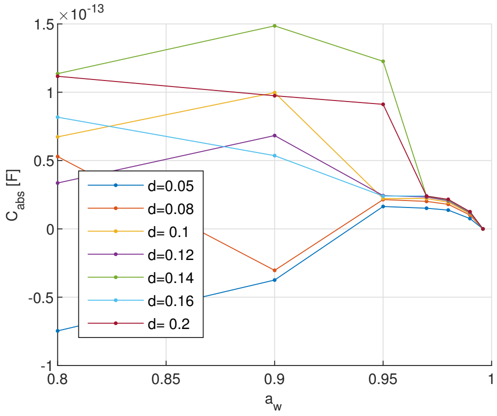

Figure S19: Absolute sensitivity with varying electrode gap of the interdigitated capacitance electrode at 1 MHz. The X axis shows the water activity and the Y axis the resulting absolute sensitivity C_{abs}. d is the electrode gap between the electrode fingers. The electrode finger width was kept at 0.1 mm.

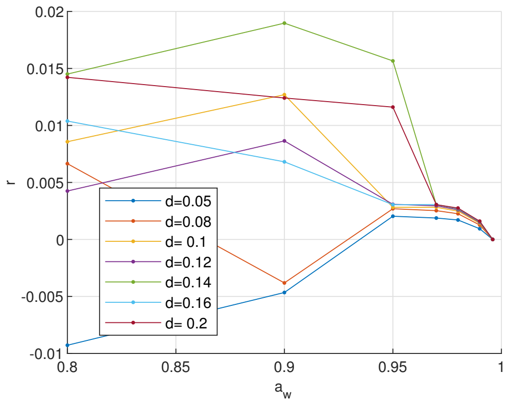

Figure S20: Relative sensitivity with varying electrode gap of the interdigitated capacitance electrode at 1 MHz. The X axis shows the water activity and the Y axis the resulting relative sensitivity r. d is the electrode gap between the electrode fingers. The electrode finger width was kept at 0.1 mm.

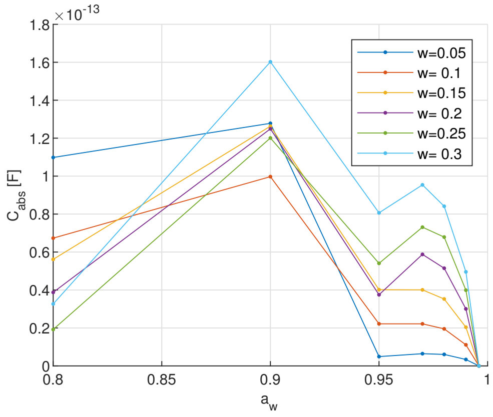

Figure S21: Absolute sensitivity with varying electrode size of the interdigitated capacitance electrode at 1 MHz. The X axis shows the water activity and the Y axis the resulting absolute sensitivity C_{abs}. w is the electrode finger width. The electrode gap was kept at 0.1 mm.

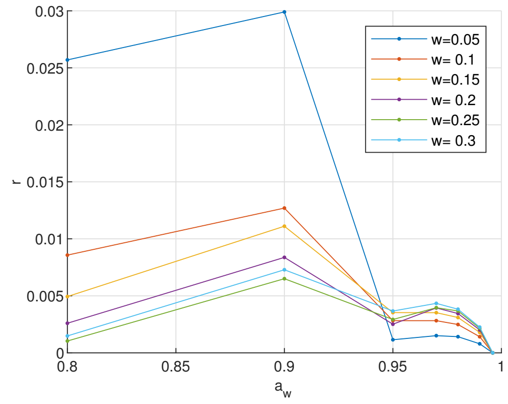

Figure S22: Relative sensitivity with varying electrode size of the interdigitated capacitance electrode at 1 MHz. The X axis shows the water activity and the Y axis the resulting relative sensitivity r. w is the electrode finger width. The electrode gap was kept at 0.1 mm.
